# Navigating the archaeal frontier: insights and projections from bioinformatic pipelines

**DOI:** 10.3389/fmicb.2024.1433224

**Published:** 2024-09-23

**Authors:** Val Karavaeva, Filipa L. Sousa

**Affiliations:** ^1^Genome Evolution and Ecology Group, Department of Functional and Evolutionary Ecology, University of Vienna, Vienna, Austria; ^2^Vienna Doctoral School of Ecology and Evolution, University of Vienna, Vienna, Austria

**Keywords:** archaea, energy metabolism, microbial dark matter, microbial ecology, microbial diversity

## Abstract

Archaea continues to be one of the least investigated domains of life, and in recent years, the advent of metagenomics has led to the discovery of many new lineages at the phylum level. For the majority, only automatic genomic annotations can provide information regarding their metabolic potential and role in the environment. Here, genomic data from 2,978 archaeal genomes was used to perform automatic annotations using bioinformatics tools, alongside synteny analysis. These automatic classifications were done to assess how good these different tools perform in relation to archaeal data. Our study revealed that even with lowered cutoffs, several functional models do not capture the recently discovered archaeal diversity. Moreover, our investigation revealed that a significant portion of archaeal genomes, approximately 42%, remain uncharacterized. In comparison, within 3,235 bacterial genomes, a diverse range of unclassified proteins is obtained, with well-studied organisms like *Escherichia coli* having a substantially lower proportion of uncharacterized regions, ranging from <5 to 25%, and less studied lineages being comparable to archaea with the range of 35–40% of unclassified regions. Leveraging this analysis, we were able to identify metabolic protein markers, thereby providing insights into the metabolism of the archaea in our dataset. Our findings underscore a substantial gap between automatic classification tools and the comprehensive mapping of archaeal metabolism. Despite advances in computational approaches, a significant portion of archaeal genomes remains unexplored, highlighting the need for extensive experimental validation in this domain, as well as more refined annotation methods. This study contributes to a better understanding of archaeal metabolism and underscores the importance of further research in elucidating the functional potential of archaeal genomes.

## 1 Introduction

Archaea, as a domain of life, has been a source of continual surprises (Cavicchioli, 2010; Jarrell et al., 2011), with ongoing discoveries helping us to understand the processes conserved in all domains of life and revealing novel types and unique features of metabolism (DiMarco et al., 1990; Weiss and Thauer, 1993; Verhees et al., 2003; Siebers and Schönheit, 2005; Thauer et al., 2008), unique structural features (Nickell et al., 2003; Moissl et al., 2005; Walsby, 2005; Matsumi et al., 2011; Zillig et al., 1981), and new genomes (Mara et al., 2023; Zhang I. H. et al., 2024), and novel lineages at the highest rank. Examples of those would be the new supergroups of archaea such as DPANN (Dombrowski et al., 2019; Zhang R. Y. et al., 2024) and Asgard (Spang et al., 2015; Zaremba-Niedzwiedzka et al., 2017; Imachi et al., 2020; Rodrigues-Oliveira et al., 2023). Moreover, the archaeal domain continuously reveals new sides to already known aspects of microbial metabolism, with novel metabolic capabilities for old enzymes, such as the case of McrA, previously a marker for methanogenesis and anaerobic methane oxidation (Friedrich, 2005), whose role in metabolism of higher carbon compounds was shown (Goenrich et al., 2004; Scheller et al., 2013; Laso-Pérez et al., 2016; Musat et al., 2024) in agreement with early *in vitro* experiments with higher carbons (Gunsalus et al., 1978), or the discovery of the metabolic potential of *Ca.* Korarchaeota for dissimilatory sulfite reduction (McKay et al., 2019). Notably, Archaea have played a pivotal role in the evolution of eukaryotes, indicating their significance in the history of life on Earth (Spang et al., 2015; Mac Leod et al., 2019; Imachi et al., 2020; López-García and Moreira, 2020; Eme et al., 2023; Spang, 2023, to name a few). The presence of eukaryotic signature proteins within Asgard genomes also led to an increased interest in the archaeal cell biology in the last few years, with a myriad of papers published on the topic (van Wolferen et al., 2022; Charles-Orszag et al., 2024; Makarova et al., 2024).

Archaea are everywhere, including the gut and skin of humans and other animals (Thomas C. M. et al., 2022; Moissl-Eichinger et al., 2017), with possibly a beneficial role. Yet, primarily due to methodological limitations (Taffner et al., 2019; Song et al., 2019), and possibly the biases in funding towards pathogens or biotechnologically relevant organisms (Lasken and McLean, 2014), the role of archaea with their host and other microorganisms remains largely unknown. Furthermore, archaea are present in plants, where, besides ammonia-oxidizing archaea (AOA) in the rhizosphere and leaves (Taffner et al., 2019; Song et al., 2019), methanogens (Taffner et al., 2019; Taffner et al., 2018) and halophilic (salt-loving) archaea can also be found (Taffner et al., 2018; Yadav et al., 2015; Al-Mailem et al., 2010). Thus, besides the impact of AOA and their role in increasing plant yield, using metagenomic sequencing techniques, indirect roles in plant growth-promoting traits, such as auxin production and production of secondary metabolites to aid against pathogens, abiotic, and biotic stress, were proposed (Taffner et al., 2018). These examples clearly show the archaeal versatile roles across different ecosystems. Whether thriving in extreme environments (*Sulfolobales, Halobacteria*) or existing in more common settings (*Nitrososphaerota*; Chaban et al., 2006), archaea remain enigmatic due to their unique adaptations and historical research biases towards the study of (pathogenic) bacteria. For instance, so far, no one really knows all enzymes involved in archaeal ammonia oxidation (Schleper and Nicol, 2010).

Since the origin of life on our planet, archaeal microorganisms continue to be fundamental to biogeochemical cycles, profoundly influencing ecosystems and environmental processes (Falkowski et al., 2008; Zhang C. et al., 2023; Qi et al., 2024; Lyons et al., 2024; Baker et al., 2020). Archaea contribute significantly to cycles involving sulfur (S; Offre et al., 2013; Neukirchen et al., 2023), nitrogen (N; Huang et al., 2021; Offre et al., 2013; Leigh, 2000), carbon (C; Boetius et al., 2000; Offre et al., 2013; Fuchs, 2011; Zhang X. et al., 2023; Justice et al., 2012; Kaster et al., 2011; Thauer et al., 1977; Thauer et al., 2008), oxygen (O; Bandeiras et al., 2005; Teske, 2018; Luo et al., 2024), iron (Fe, Dong et al., 2021; Auernik and Kelly, 2008; Malik and Hedrich, 2022), and arsenic (As; Zhang C. et al., 2023; van Lis et al., 2013) across various habitats. Their metabolic versatility and resilience in extreme environments make archaea indispensable for maintaining the equilibrium of these elemental cycles, impacting nutrient availability, greenhouse gas emissions, and overall ecosystem health (Falkowski et al., 2008; Zhang X. et al., 2023; Qi et al., 2024; Lyons et al., 2024).

Methanogens, halophiles, thermophilic *Euryarchaeota* and *Thermoproteota* have become valuable model systems in molecular biology and biotechnology (Allers and Ngo, 2003; Kletzin, 2007; Soppa, 2006; Leigh et al., 2011; Costa and Whitman, 2023; De Lise et al., 2023; Pfeifer et al., 2021; Aparici-Carratalá et al., 2023), and currently these four groups of archaea boast well-established genetic systems. This advancement renders them ideal for use as model organisms and facilitates the expanded exploration of the functions of archaeal genes. However, the biotechnological potential of recently discovered archaeal lineages remains to be explored.

At the heart of archaeal diversity lies their genomic repertoire, comprising a finite set of protein building blocks, organized into pathways that facilitate biochemical reactions. One prominent example is methanogenesis, a pathway wherein certain archaea produce methane through anaerobic metabolism, essential for carbon cycling in environments like wetlands, alkaline hydrothermal vents, and animal digestive tracts (Angle et al., 2017; Jones et al., 1983; ver Eecke et al., 2012; Thomas P. D. et al., 2022), and that is proposed to have had an important role at the origin of Life (Martin and Russel, 2007). Additionally, many archaea engage in chemolithotrophy, deriving energy by oxidizing inorganic compounds such as hydrogen, sulfur, or iron (Thauer et al., 1977; Edwards et al., 2000; Pereira et al., 2011; Colman et al., 2020).

With the advent of metagenomics, many novel lineages have been discovered, for which mainly only metagenomic information is available for metabolic reconstructions using functional annotation pipelines. However, most of these are biased toward bacterial knowledge, with archaeal proteins many times falling out of the established cutoffs due to their natural diversity. Thus, it is important to assess how much of this diversity can be retrieved semi-automatically using functional annotation pipelines. Moreover, this approach can, in a systematic way, pinpoint gaps in knowledge, driving for the experimental characterization of archaeal proteins, as well as a redefinition of model design. Several studies regarding microbial dark matter, particularly Archaea, have been put forward, where the ratios vary between 30 and 80% (e.g., Makarova et al., 2019; Rinke et al., 2013; Jiao et al., 2020). More recently, deep learning was applied to genomes to get insights from microbial dark matter, showing how relevant the characterization of microbial dark matter is (Hoarfrost et al., 2022).

The question put out in this paper is: do existing automated prediction tools perform as well at assigning gene functions to archaea as to bacteria? Thus, to deepen our understanding of archaeal biology and metabolism, we performed a comprehensive mapping of genomic data from 2,978 archaeal genomic assemblies, belonging to 27 phyla (including unclassified Archaea) and compared the results to the ones obtained from a similar number of bacterial assemblies (175 phyla). This initiative aims to assess the gaps in predicted knowledge about archaea, and compare it to bacteria. Through systematic exploration and analysis, we can pinpoint gaps in predictive knowledge and guide experimental studies with the aim of further understanding the diverse metabolic capabilities and ecological significance of archaea.

## 2 Materials and methods

### 2.1 Genomic dataset

A subset of our in-house dataset (over 190,000 genomes, 2,629 of which are archaeal; downloaded from NCBI in November 2019 with two *Acidianus ambivalens* and one *Ca. Lokiarchaeum ossiferum* assemblies added later; Supplementary Table 1; Rodrigues-Oliveira et al., 2023) was created by filtering these assemblies by completeness and contamination (calculated using the “Rinke method,” Rinke et al., 2013), excluding all with contamination >20%. In addition, assemblies containing more than 10% contamination were excluded unless there were only two or less representatives per genus. Assemblies with low contamination were filtered for completeness: if the genus had more than two representatives, those with <40% completeness were excluded. In case of DPANN archaea, genomes were excluded only if their completeness was <20% and they had more than one representative per genus. To capture the recent sequenced diversity of archaea, additional genomes were downloaded from JGI (1,731 genomes). For this study, the total of 2,978 archaeal assemblies, belonging to 27 phyla (incl. “unclassified Archaea”) were obtained. In addition, a set of 3,235 bacterial genomes, belonging to 175 phyla (2 representatives per genus) used for comparison (Supplementary Table 1).

### 2.2 Functional annotation

The 2,978 archaeal and the 3,235 bacterial genomic assemblies were functionally annotated using KEGG HMM profiles (version 2024-02-28, Kanehisa and Goto, 2000; using HMMER version 3.4, hmmer.org). The resulting hits were filtered first by cutoffs provided by KEGG for each model, and second, by lowering the KEGG cutoffs by 20% for most models, except for cytochrome *bc1* complex models, where the previously established in-house cutoffs were used (Supplementary Table 2). The cutoffs were lowered to account for the fact that the standard KEGG cutoffs do not always work for the archaeal sequences. If no KEGG cutoff was provided for a model, a cutoff of 50 was used to ensure the hits for these KOs of acceptable quality were still included in the analysis. The KEGG name, module and pathway information was mapped to the resulting annotations.

The dataset was additionally annotated using Interproscan (version 5.66–98.0; Jones et al., 2014), which includes the following databases: CDD (NCBI Conserved Domain Database; Lu et al., 2020), PFAM (Mistry et al., 2007), Gene3D (Lees et al., 2012), PANTHER (Thomas C. M. et al., 2022), SUPERFAMILY (Gough and Chothia, 2002; Wilson et al., 2009), ProSitePatterns and ProSiteProfiles (Expasy Prosite; Sigrist et al., 2013), NCBIfam (also known and further referred to as TIGRFAM, Li et al., 2021), FunFam (Sillitoe et al., 2013), Hamap (Pedruzzi et al., 2015), PIRSF (Wu et al., 2004), Coils (Lupas et al., 1991), MobiDB-lite (Necci et al., 2021), SMART (Letunic et al., 2021), PRINTS (Attwood et al., 2012). PANTHER annotations were further filtered to eliminate uncharacterized proteins, domains of unknown functions (DUF) and annotations solely as “membrane protein” or “conserved protein.”

Furthermore, the archaeal genomes were annotated using the information obtained from DiSCo (Neukirchen and Sousa, 2021). Diamond Blast searches were also performed to assign arCOG (Makarova et al., 2015; Liu et al., 2021) classification to all genomes, by selecting best hits using as cutoffs >= 25% identity and E-value of <= 0^−10^.

### 2.3 Sequence classification into “characterized” and “uncharacterized”

The resulting annotated hits were split into “characterized” and “uncharacterized” sets using the following strategy (as described in Supplementary Figure 1): If the sequence has a KEGG annotation with a KEGG pathway annotation “Function unknown,” then it is classified as “uncharacterized”; if the KEGG pathway annotation is different, then the sequence is classified as “characterized.” If the sequence has no KEGG annotation, then the PANTHER annotation is checked. If a PANTHER annotation is present and it is not in the curated list of uncharacterized PANTHERs (Supplementary Table 3), the sequence is classified as “characterized”; if it is in the list, the sequence is “uncharacterized.” If no PANTHER annotation is present, then the NCBIfam (TIGRFAM) annotation is checked. If a TIGRFAM annotation is present in the curated list of “uncharacterized TIGRFAMs” (Supplementary Table 3), then the sequence is classified as “uncharacterized”; otherwise, it is assigned as “characterized.” If no TIGRFAM annotation is available, the Hamap annotation is checked: if it is present, the sequence is classified as “characterized,” otherwise, it is classified as “uncharacterized.”

Sequences without any annotations were automatically classified as “uncharacterized.” The order of the steps is partially arbitrary, and, starting with KEGG annotations, the classification steps can be run in a different order if preferred. The reason for selecting KEGG as initial step is three-fold: KEGG is a widely used database in which metabolic maps were constructed manually, and KEGG orthology is usually based on characterized enzymes or proteins. Lastly, KEGG provides modules and higher classifications of metabolism which are of interest for this analysis. This pipeline is available at https://github.com/valkaravaeva/protein_classification_tool.

### 2.4 Analysis of “uncharacterized” sequences

The mean, median, maximum, and minimum numbers of uncharacterized sequences were calculated per taxon (in percent of uncharacterized vs. total CDS per genome) at a phylum level. The PFAM annotations of archaea were analyzed, in terms of most common occuring domains per taxon (supergroup or phylum). The values per lineage were plotted as a boxplot using “ggplot2” package in R.

### 2.5 Comparison between “uncharacterized” archaeal sequences and ArCOGs

ArCOG annotation was used as a comparison to the pipeline in terms of uncharacterized proteins. Sequences without arCOG annotation or with the functional model belonging to “S_Function_unknown,” “4_Poorly_Characterised” and “R_General_Function_Prediction_only” category, or having no category were classified as “uncharacterized.” The mean, median, max, and min percentages per phylum of “uncharacterized” sequences based on arCOGs were computed and plotted, as described in section 2.4. The intersection between uncharacterized proteins between both methods as well as the method specific were analyzed. The values per lineage were plotted as a boxplot using “ggplot2” package in R.

### 2.6 Analysis of “characterized” sequences

The set of “characterized” sequences was analyzed in terms of KEGG module completeness (computed in percent; accounting for alternative KOs and for complexes—see pipeline documentation and files at https://github.com/valkaravaeva/protein_classification_tool and additional files at FigShare: 10.6084/m9.figshare.25782123). Briefly, per assembly, each module, including the different alternatives for each step was considered complete if there were identified proteins for at least one route (100%). If one or more proteins were missing, the ratio of identified proteins versus the number of pathway proteins needed was calculated and multiplied by 100. In the case of complexes, a similar approach was taken, in this case, using the number of identified subunits as numerator. This information was used to analyze the metabolic potential of each genome and *a posteriori*, aggregated by phylum. Further analyses were focused on cofactor biosynthesis and energy metabolism. For this, KOs of selected gene markers were used to represent types of energy metabolism. In addition, in specific cases, existing KEGG modules were manually modified, or created, by either joining several modules for the same pathway or complex, or, as in case of riboflavin biosynthesis, since no KEGG module for the archaeal version is available, by using the BioCyc database entry for *M. jannaschii* (and corresponding KO annotations; Karp et al., 2019). Completeness of these manual modules was assessed in the same way as for original KEGG modules. Completeness of selected modules was plotted as a stacked barchart using “ggplot2” package in R. Taxonomic distribution of selected marker genes was plotted as a heatmap using R package “Pretty heatmaps” (https://cran.r-project.org/web/packages/pheatmap/pheatmap.pdf) and beautified in Inkscape.

## 3 Results

**Figure 1:**
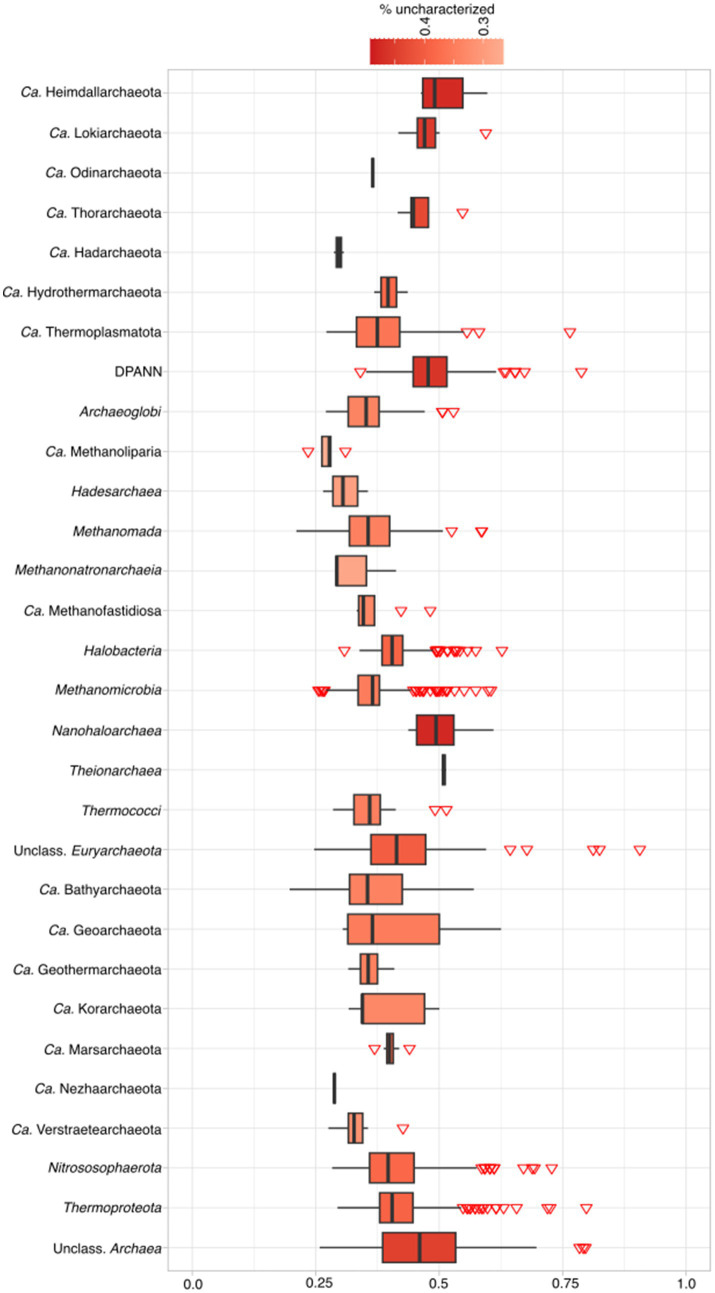
Percentage of archaeal unclassified proteins according to the pipeline classification per phylum. For complete taxonomic information see Supplementary Table 1. For exact percentages, see Supplementary Table 4.

**Figure 2:**
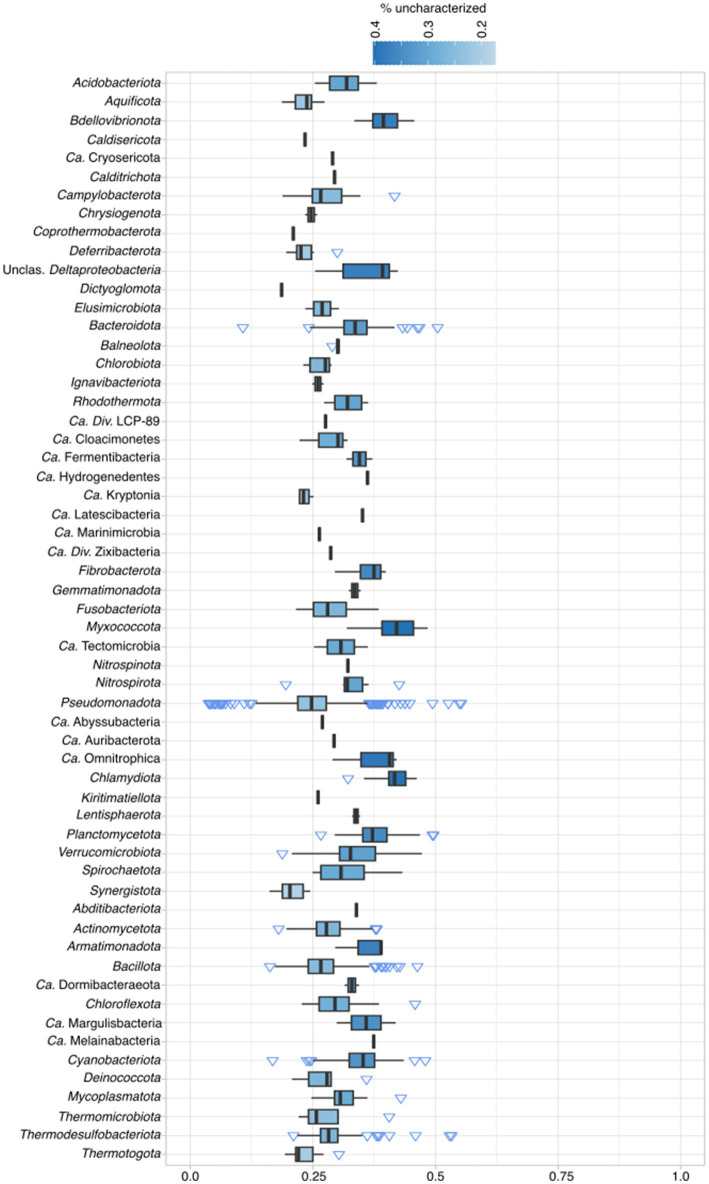
Percentage of unclassified bacterial proteins according to pipeline classification (see Materials and Methods) per phylum. For complete taxonomic information see Supplementary Table 1. For exact percentages, see Supplementary Table 4.

To determine how much of archaeal proteomes fall into the category of uncharacterized, a pipeline with several different steps was employed (see Supplementary Figure 1 and Materials and Methods). In total, 2,451,799 (40.7%, lowered KEGG cutoffs) out of 6,029,057 of proteins fall into the uncharacterized category, from where newly discovered lineages, such as *Ca.* Heimdallarchaeota and *Ca.* Woesearchaeota, have a mean of ~50% of proteins classified as uncharacterized (Figure 1; Supplementary Table 4). Within Archaea, 16 out of 27 groups (59%; including unclassified Archaea) have more than 40% of its proteins classified as uncharacterized, and only in two groups this ratio falls shortly below 30%. The average of uncharacterized proteins across all analyzed archaeal genomes is 42%. When examining the percentage of uncharacterized proteins per phylum, in bacteria, only 45 out of 175 phyla (25%) have more than 40% uncharacterized proteins (Figure 2, Candidate phyla in Supplementary Figure 2). When comparing model organisms from both domains, and even excluding *E. coli* (12%), there are 31 bacterial phyla where at least one organism has less than 25% uncharacterized proteins. In contrast, among archaea, only the *Candidatus* Bathyarchaeota and *Euryarchaeota* have at least one assembly with less than 25% uncharacterized proteins. Moreover, while lowering KEGG model cutoffs induced a change in the number of archaeal unclassified proteins, it did not affect the number of uncharacterized bacterial proteins, indicating that the models are optimized for this domain (Supplementary Table 4).

The two assemblies of the archaeal group with lowest median percentage of uncharacterized proteins, *Ca.* Nezhaarchaeota, have a low number of proteins (fewer than 1,700) and have completeness scores of 88 and 93%, and contamination of 0.6 and 4.3%, respectively. Their reduced genome, potentially associated with a symbiotic lifestyle, could explain the median percentage of uncharacterized proteins being below 30%. In any case, this value is still roughly three times the one found for model bacteria. This pinpoints the problems in reconstructing the metabolism of newly sequenced archaeal lineages.

Within the 2,451,799 unclassified proteins, 33.8% have PFAM annotation, while 66.2% (1,622,446) lack any annotation. Remarkably, with the exception of *Ca.* Nezhaarchaeota, *Ca.* Hadarchaeota, and *Ca.* Verstraetearchaeota, where 46.6, 55.4, and 59.0% of uncharacterized proteins lack PFAM annotations, respectively, all other archaeal phyla exhibit over 60% of uncharacterized proteins devoid of PFAM annotations, leaving even their domains unidentified. The uncharacterized proteins with PFAM domains have their annotations spread over 16,689 different PFAM entries, from where 3,725 correspond to domains or proteins with unknown functions. The remaining 12,964 PFAM domains are found in 829,353 proteins (33.8%), with 6,602 present in less than 10 proteins.

Notably, the prevalent PFAM among those with annotations is the PIN domain, characterized by three conserved acidic residues but limited conservation otherwise, which in eukaryotes is associated with ribonucleases (Arcus et al., 2011), and in prokaryotes, it is a component of the toxin-antitoxin system (TA; Arcus et al., 2011). In fact, ~44% of those PIN domains are in the proximity of genes annotated as nosB or Vap, being potentially part of a toxin-antitoxin system (TA, Arcus et al., 2011; Bunker et al., 2008) or close to CRISPR-Cas systems. The remaining PIN domains are in the vicinity of enzymes, ribosomal proteins or other uncharacterized genes. The large superfamily of PIN domain proteins was divided into families (Matelska et al., 2017) and a role as endo/exonucleases and/or part of the defense arsenal proposed (Matelska et al., 2017). The second most frequent domain is “LexA-binding inner membrane-associated putative hydrolase,” which is found in phospholipases and in proteins belonging to the SOS network, which rescues cells from DNA damage (Zhang and Lin, 2012). The third most frequent domain is the “halobacterial output domain 1,” which is specific for haloarchaea and haloviruses, and possibly involved in regulatory processes (Galperin et al., 2018). The fourth most frequent domain overall is the helix-turn-helix domain, usually found in transcriptional regulatory proteins and involved in DNA binding that, in some cases, can also be found in multidomain proteins for nucleotide recruitment, or involved in protein–protein interactions (Menon and Lawrence, 2013). In fact, among the 20 most frequent PFAMs, additional DNA-binding annotations emerge, including two winged helix-turn-helix domains, and the transcriptional regulator TrmB (Supplementary Table 5). TrmB, a sugar-specific transcriptional regulator of the trehalose/maltose ABC transporter from the hyperthermophilic archaeon *Thermococcus litoralis*, was previously characterized (Lee et al., 2003). Also, the *H. salinarum* reactive oxidative species regulator (RosR arCOG00006), which was experimentally characterized (Sharma et al., 2012) and whose crystallographic structure is available (Kutnowski et al., 2019) is annotated as hypothetical protein and has no annotation within KEGG. These examples point to the misidentification of archaeal regulatory networks, in some cases, due to lack of models, in others, due to lack of characterization, as in the case of the ArsR/SmtB family (Lemmens et al., 2019). Moreover, the 6,602 PFAMs identified in fewer than 10 uncharacterized proteins, underscore the vast potential for innovation and diversity within this domain (Supplementary Table 5). Additionally, when examining proteins typically associated with metabolism, over 450 PFAMs (excluding radical SAM enzymes), corresponding to approximately 14,000 proteins, have annotations indicating the presence of hemes, FAD, NADH, molybdopterins, iron–sulfur clusters, oxidoreductases, or quinone-binding. This suggests that a portion of the archaeal metabolism remains not fully understood. A typical example would be the case of molybdopterin enzymes, which are ubiquitously present in prokaryotes, though the function of some is not known (Wells et al., 2020; Roy and Adams, 2002; Bevers et al., 2005).

The synteny analysis of unclassified proteins has shown that in 2,866 assemblies (96.2% of the dataset), there is at least one stretch of five or more genes without any available annotation (no PFAM). This number is even larger when considering the existence of pseudogenes in between uncharacterized ones. As a result, significant portions of the archaeal genomes remain without biological predictions, due to various factors such as the absence of models, assembly artifacts such as technical fusions, fissions or erroneous sequences (Padalko et al., 2024), inadequate CDS predictive methods for archaea (Dimonaco et al., 2022; Meng et al., 2022), or simply lack of biological knowledge. Notably, the uncharacterized genes within these regions are not necessarily involved in the same biological process, as genomic rearrangements frequently occur within genomes (Bobay and Ochman, 2017; Tillier and Collins, 2000; Darmon and Leach, 2014). When focusing on uncharacterized proteins for which PFAMs are available, particularly those which could, *a priori*, give some indication regarding energy metabolism, we observe that, for some cases, the uncharacterized protein’s PFAM agrees with the surrounding genes, e.g., PF00507 and PF00420 NADH–ubiquinone/plastoquinone_oxidoreductase, _chain_3 and 4 L from complex I surrounded by other Complex I subunits (Supplementary Table 6). This indicates that their nonidentification by other methods might be due to the model not accounting for the full range of sequence diversity. In this case, the full predicted complex could, with thorough analysis, be recovered. In other cases, putative complexes have no attributed annotation except PFAM, making their identification more difficult. Those are the cases, for instance, for Complex IV subunits in proximity of each other in known aerobic organisms, such as *Halobacteria*, where both subunit I and subunit II (the catalytic ones) are found within a distance of four or less genes devoid of further annotations. While subunit II tends to be a transmembrane short protein, devoid of cofactors (for exceptions see Pereira et al., 2011; Murali et al., 2022), subunit I is composed of a conserved set of 12 transmembrane helices, containing the ligands for the low-spin heme and for the binuclear center, composed of a high-spin heme and a copper ion. This subunit, outside of the HCO family, has homology only with nitric oxide reductases (Pereira et al., 2011). Thus, the subunit I fold is specific to these enzymes, and, possibly due to sequencing artifacts, falls below the usual model cutoffs. In this way, the complex IV, previously described to be present in *Ca.* Heimdallarchaeota assemblies (Spang et al., 2019; Bulzu et al., 2019), could not be identified. Even though *Halobacteria* thrive in oxic environments (Grant and Ross, 1986; Oren, 1994; Oren and Litchfield, 1999; Cui and Dyall-Smith, 2021), and several Asgard assemblies have been obtained from oxic conditions (Bulzu et al., 2019), additional experimental characterizations are necessary to ascertain whether these “HCOs” can reduce O_2_, utilize alternative terminal electron acceptors, or even function effectively.

**Figure 3:**
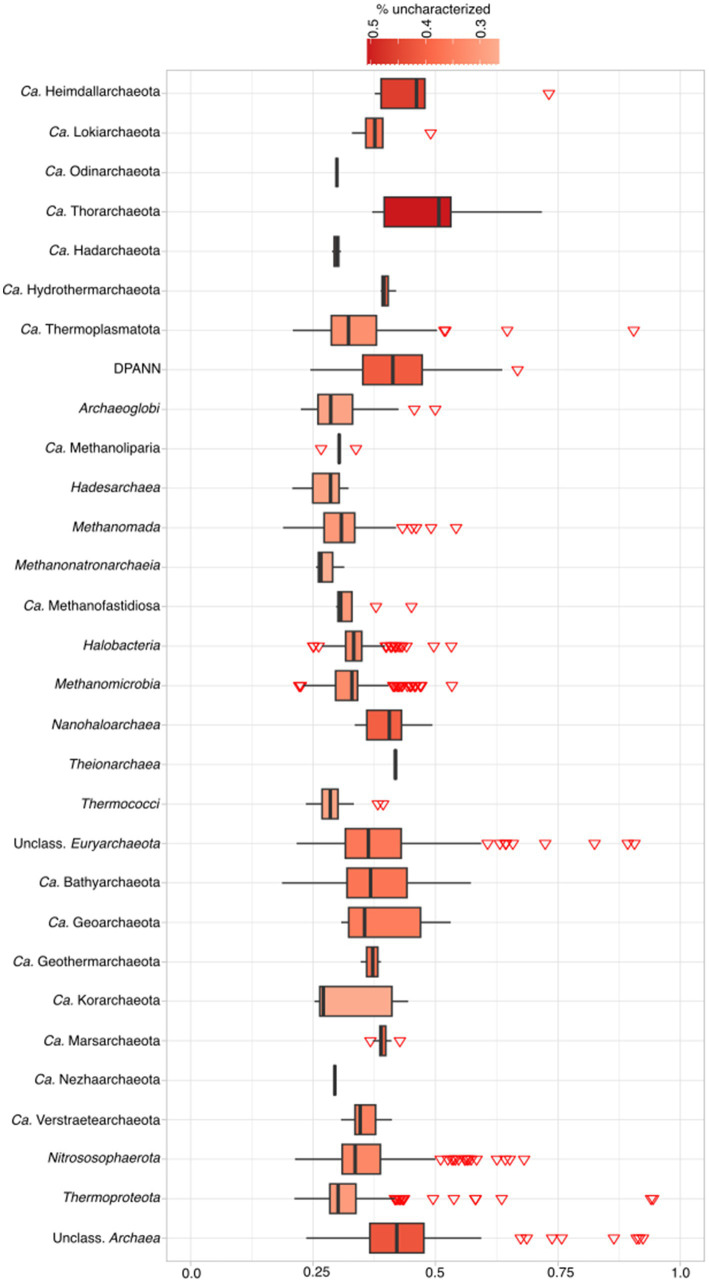
Percentage of unclassified archaeal proteins according to arCOG classification per phylum. For complete taxonomic information see Supplementary Table 1. For exact percentages, see Supplementary Table 4.

**Figure 4:**
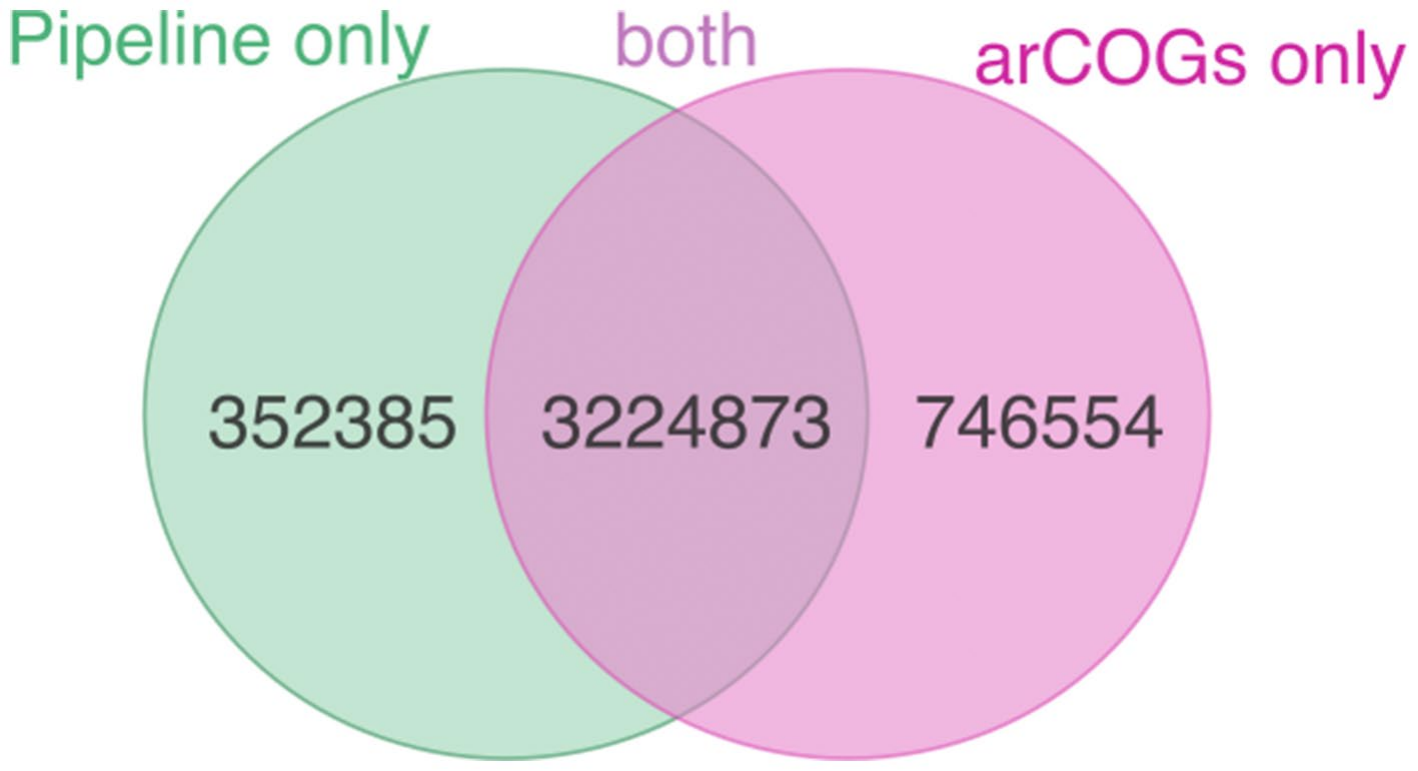
Number of proteins classified as “characterized” only by pipeline (green), only by arCOGs (pink), and by both methods (intersection).

We compared the results of our pipeline (available at https://github.com/valkaravaeva/protein_classification_tool) with the functional classification given by arCOGs (Makarova et al., 2015; Liu et al., 2021), a tool developed specifically for the identification of archaeal clusters of orthologous groups. Depending on the lineages, either arCOG (18; Figure 3) or our pipeline (4) has less uncharacterized proteins (Figure 1), with 5 phyla achieving similar results (differences below 1%). However, overall, arCOG outperforms our pipeline by identifying approximately 350,000 fewer uncharacterized proteins in total (see Figure 4). This advantage is also evident in lineages with lower overall numbers, such as *Ca.* Woesearchaeota and *Ca.* Heimdallarchaeota, which have mean proportions of 47 and 48% “unclassified” proteins, respectively, compared to 51% for both lineages using our pipeline. Additionally, four out of 27 archaeal phyla show a ratio of unclassified proteins just below 30% using arCOGs, whereas 9 out of 27 have more than 40% uncharacterized sequences.

The large majority of the proteins only classified by arCOGs belong to informational (transcription, translation, replication), defense, mobilome and cellular processes (74%), in agreement with the effort of the authors of arCOGs in improving those modules (Makarova et al., 2015; Liu et al., 2021) combined with the underdevelopment of KEGG in those modules. On the other hand, within the over 350,000 proteins only annotated by our pipeline, and focusing on the ones with KEGG annotations (corresponding to 45% of the proteins only annotated by the pipeline), 62% belong to the metabolism category, with signaling and cellular processes (20%) and informational processes (17%) as following categories. Among the proteins from metabolism are, for instance, 2000 involved in methane metabolism, including several acetyl-CoA synthase and formylmethanofuran dehydrogenase subunits, over 8,300 proteins involved in energy metabolism such as sulfide:quinone oxidoreductases (Brito et al., 2009), thiosulfate:quinone oxidoreductases (Müller et al., 2004), and V/A-type H+/Na+-transporting ATPase subunits, from where the *Methanobrevibacter ruminantium* complex was experimentally validated (McMillan et al., 2011). Therefore, to avoid running both approaches and to standardize the data, the choice of annotation strategy should depend on the specific goals of the study.

However, arCOGs are built from a graph method in which, due to, e.g., gene losses, paralogues can be grouped together. Moreover, there is no relationship between KOs and arCOGs, which renders the mappings of pathways using arCOGs for a database as large as the one used in this paper, an “Herculean” task. Thus, we continued the analysis using KEGG annotations.

The functional classification of archaeal proteins allows to reconstruct their metabolic potential and pinpoint possible gaps within pathways to be further experimentally characterized. Using KEGG modules combined with a strategy to count for their completeness (see Materials and Methods), the full reconstruction of the metabolism of 2,978 genomes is presented in Supplementary Table 7. Out of the 479 modules, 115 had no hit for archaeal proteins and only 20 were found to be complete or at least 75% complete in more than 50% of the assemblies in our dataset. These mainly correspond to the building blocks of life, such as nucleotides, amino acids and cofactors biosynthesis, ATP synthesis, lipid biosynthesis as well as carbon metabolism. The rationale behind setting a 75% completeness threshold includes instances where, despite adjustments, specific module components remain elusive. This also accounts for possible assembly incompleteness. Within bacteria, only 44 modules were not present in any of the genomes, and 60 modules found in more than 50% of the bacterial assemblies. The modules specific for bacteria are, for instance, gamma-aminobutyric acid (GABA) biosynthesis, a pathway present in several bacteria (Iorizzo et al., 2023) that has homologues within archaea which perform different functions (Tomita et al., 2014; Falb et al., 2008), or anoxygenic photosynthesis, a process that is absent in archaea (Hohmann-Marriott and Blankenship, 2011). Other modules address specific bacterial systems, such as antibiotic resistance or the synthesis of secondary metabolites unique to certain lineages.

In addition, several KEGG modules were modified to fill in gaps regarding archaeal metabolism not included in the original modules. For instance, in the cobalamin biosynthesis module, the decision to include CbiX, a homologue of CbiK that in some organisms performs the same reaction (Raux et al., 2003), was made to enhance the module’s completeness, since this protein was initially absent. However, this adjustment, combined with lowered cutoffs, did not result in the increase of completeness of this module as expected, since there was no identification of other expected proteins within the module, such as the adenosylcobinamide kinase/adenosylcobinamide-phosphate guanylyltransferase CobP/CobU (K02231) because archaea do not have CobP/CobU but rather use CobY (K19712; Rodionov et al., 2003), which is not a part of the KEGG cobalamin biosynthesis modules. Even considering that some archaea might not have cobalamin biosynthesis, this scenario highlights the difficulty of defining a cutoff that accurately reflects the presence of all essential components, especially in complex biosynthetic pathways. Moreover, not all of complexes are part of KEGG modules. This is the case of the Ech and Ehb membrane hydrogenases (Marreiros et al., 2013; Marreiros et al., 2016) present in many methanogens, or the thiol:fumarate reductases (Heim et al., 1998), a complex whose subunits are homologous to the catalytic subunits of Complex II (Lancaster, 2002; Karavaeva and Sousa, 2023). Another problem is the existence of several modules for the same complexes, as, e.g., in the cases of succinate dehydrogenases/fumarate reductases, heme-copper oxygen reductases, and the *bc*1 complex. This leads to the existence of many archaeal complexes that have chimeric classifications according to KEGG modules, i.e., one subunit being part of one module and the other(s) belonging to another module of the same complex. This leads to modular incompleteness and hinders the usage of KEGG modules as a proxy of archaeal metabolism. For the cases mentioned above, we considered the module present if the subunits were identified, regardless of the KO module classification, meaning KEGG modules were merged, and different possible KOs would represent the same subunit. Completing this information with TIGRFam/NCBIFam and BioCyc information for selected modules (as described in Materials and Methods) led to an increase in module and pathway completeness. Still, in most of the cases, modules fall below the 75% completeness cutoffs (Supplementary Table 7). These results suggest that our understanding of the metabolic diversity and the distribution of biosynthetic pathways among archaea is still not included into databases, and the known existing gap between Bacteria and Archaea knowledge is even more pronounced at the level of automatic annotations.

Looking in detail to the different pathways for coenzymes and cofactors biosynthesis, we can observe that regarding heme biosynthesis in archaea (Supplementary Figure 3), the siroheme-dependent route is the most widely distributed, with the coproporphyrin-dependent pathway found to be complete in some *Halobacteria*, as already described (Dailey et al., 2017), as well as in one genome of *Ca.* Hydrothermarchaeota. Interestingly, within *Ca.* Heimdallarchaeota and some unclassified Euryarchaeota, the protoporphyrin-dependent heme biosynthesis was found. *Ca.* Heimdallarchaeota organisms have a mitochondrial-like electron-transport chain, being able to respire oxygen (Zaremba-Niedzwiedzka et al., 2017). This is not found in the majority of the other Asgard lineages and might be the result of HGT events. Since *Ca.* Heimdallarchaeota is also one of the few archaeal groups with protoporphyrin-dependent heme biosynthesis, this pathway might also have been acquired by HGT. Previously, several studies have reported on large events of interdomain HGT for archaea (Koonin and Wolf, 2008; Nelson-Sathi et al., 2012; Nelson-Sathi et al., 2015), and *Ca.* Heimdallarchaeota might be one of these cases. Of note, within our dataset, many other archaea were found to contain partial protoporphyrin-dependent heme biosynthesis pathways. However, this module also contains the universal tetrapyrrole biosynthesis part, common to the biosynthesis of all tetrapyrroles cofactors (heme, cobalamin, siroheme, F_430_) that are all present in Archaea.

Regarding cobalamin biosynthesis, a full pathway is found in *Ca.* Thermoplasmatota, *Archaeoglobi*, *Ca.* Methanoliparia*, Methanomada, Methanonatronarchaeia, Halobacteria, Methanomicrobia*, *Ca.* Marsarchaeota*, Nitrososphaerota, Thermoproteota*, and unclassified Archaea. However, in most of these lineages, there are genomes that contain only a partial pathway, due to either not passing the cutoffs (especially in the case of CbiJ) or having no KO annotation for a fused protein (e.g., fusions of CbiK/CbiX chelatase and HmbS/HemC in certain *Archaeoglobi* genomes have only the KO annotation for the last protein). Fusion and fission events are a process common in Archaea, as shown in recent large-scale analysis (Padalko et al., 2024).

Complete pathways for the biosynthesis of menaquinone were found in *Ca.* Thermoplasmatota*, Archaeoglobi*, DPANN, *Thermoproteota, Methanomicrobia*, and unclassified Archaea (Supplementary Figure 4). However, only in *Archaeoglobi* and *Ca.* Hydrothermarchaeota, they were present in the majority of the taxon assemblies. The presence of menaquinone in Archaea has been previously reported for *Thermoproteus tenax* (Thurl et al., 1985). As expected, no archaeal organisms have the complete pathway for ubiquinone biosynthesis. However, many have partial pathways, indicating the presence of several enzymes, homologous to those involved in ubiquinone biosynthesis. Within Archaea, besides menaquinones, several organisms use Caldariella (Schäfer et al., 2002) or sulfoquinone (Elling et al., 2016) as main quinone. Since the biosynthesis of these alternative quinones remains, to our knowledge, not fully resolved, it is not clear if the ubiquinone biosynthesis homologues found in those lineages might play a role in other quinone biosyntheses, and those are good candidates for further experimental validations.

**Figure 5:**
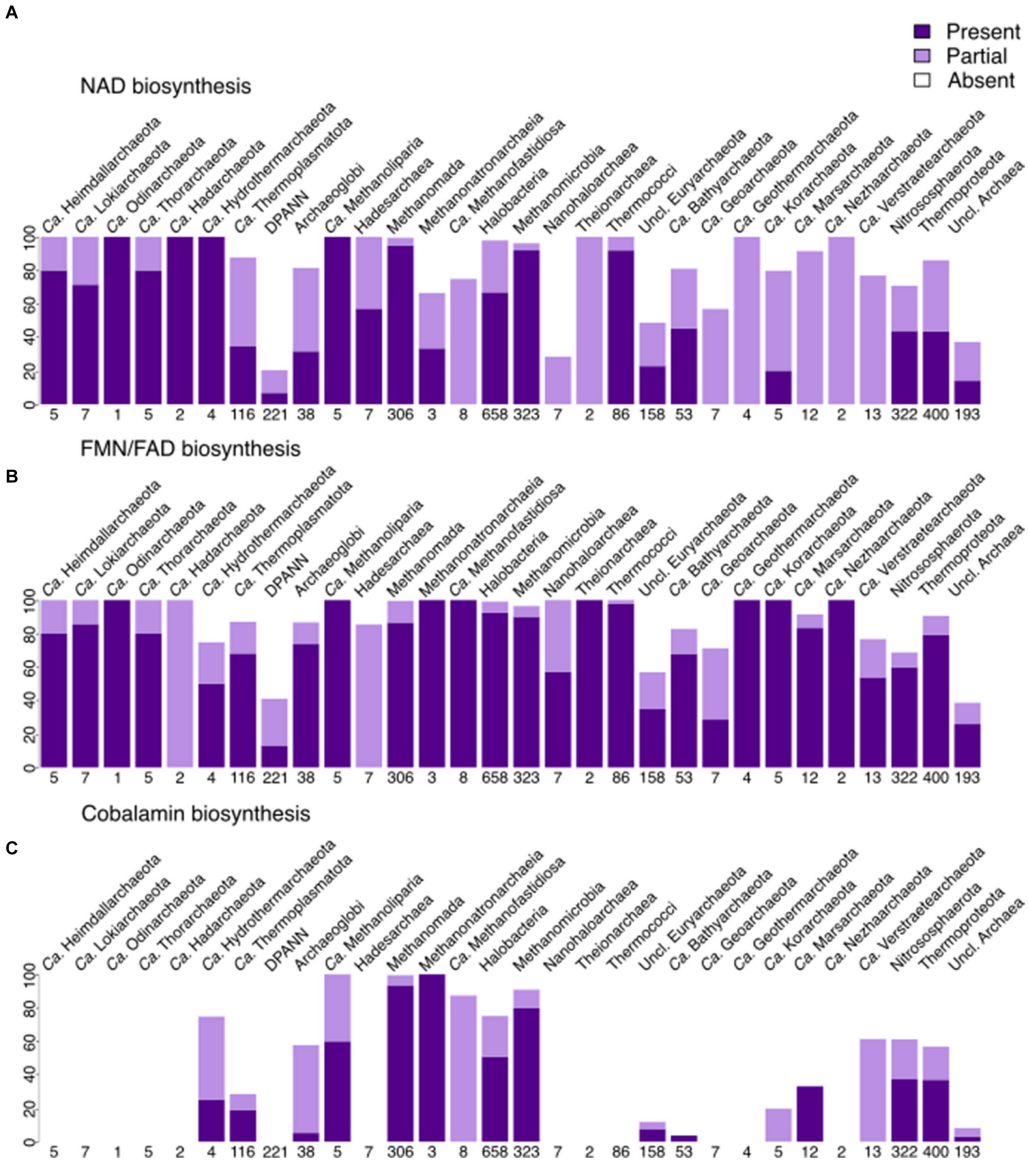
Presence of cofactor biosynthesis in archaea (per phylum for most lineages, per class for Euryarchaeota, with Methanobacteria, Methanococci, and Methanopyri grouped into the Methanomada supergroup), based on modified KEGG modules. **(A)** Presence of NAD biosynthesis (via both Tryptophan and Aspartate). **(B)** Presence of FMN + FAD biosynthesis. **(C)** Presence of cobalamin biosynthesis (excluding the lower ligand synthesis). Dark purple indicates that the full module is present, light purple marks the presence of the incomplete module, white shows the absence of the module in a lineage.

**Figure 6:**
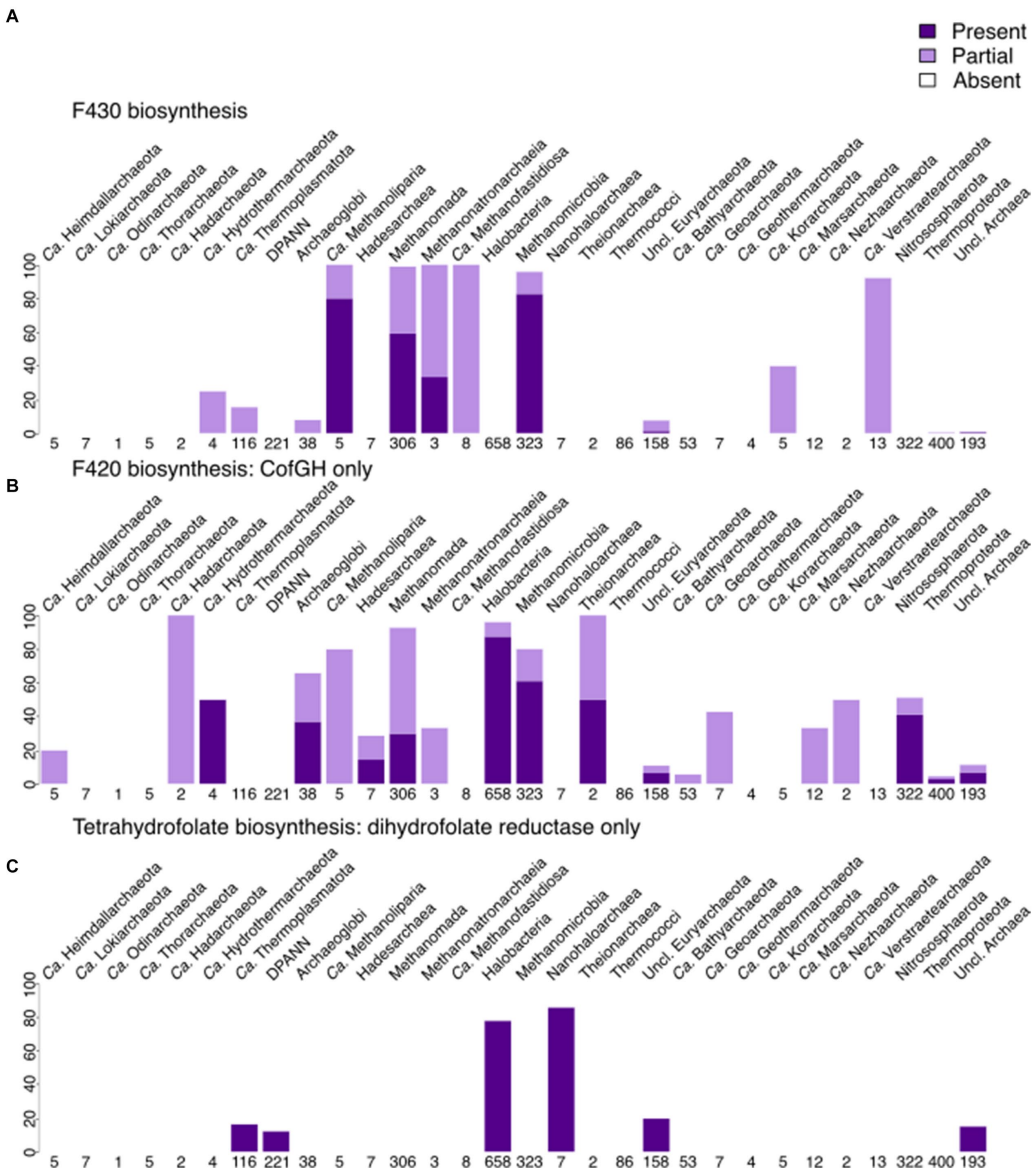
Presence of cofactor biosynthesis in archaea (per phylum for most lineages, per class for Euryarchaeota, with Methanobacteria, Methanococci, and Methanopyri grouped into the Methanomada supergroup), based on modified KEGG modules. **(A)** Presence of F_430_ biosynthesis. **(B)** Presence of F_420_ biosynthesis (includes only CofG + CofH as markers). **(C)** Presence of tetrahydrofolate biosynthesis (includes only dihydrofolate reductase as a marker). Dark purple indicates that the full module is present, light purple marks the presence of the incomplete module, white shows the absence of the module in a lineage.

Contrary to menaquinone biosynthesis, riboflavin (incl. FMN/FAD; Figure 5 and Supplementary Figure 4) biosynthesis is found to be partially present in many archaeal lineages, being complete within several lineages, such as *Archaeoglobi, Halobacteria, Methanomada, Theionarchaea, Nitrososphaerota*, and *Thermococci*. FMN/FAD biosynthesis enzymes are present in all lineages, including DPANN. Even with our improved module for FAD biosynthesis, we noticed that the enzyme(s) responsible for converting GTP to 2,5-Diamino-6-(1-D-ribosylamino)pyrimidin-4(3H)-one-5′-phosphate are absent in most archaea, indicating a gap in knowledge that possibly only experimentalists can fill. The biosynthesis of F_430_ is, as expected, present in several methanogenic groups (Figure 6) being less spread than the biosynthesis of F_420_ that besides methanogens, is also found in *Archaeoglobi*, *Ca.* Heimdallarchaeota, *Ca.* Lokiarchaeota, *Halobacteria* and *Theionarchaea*. The dihydrofolate reductase, used as a marker for folate biosynthesis, is mainly found in most assemblies from *Halobacteria* and the related group *Nanohaloarchaea*.

**Figure 7:**
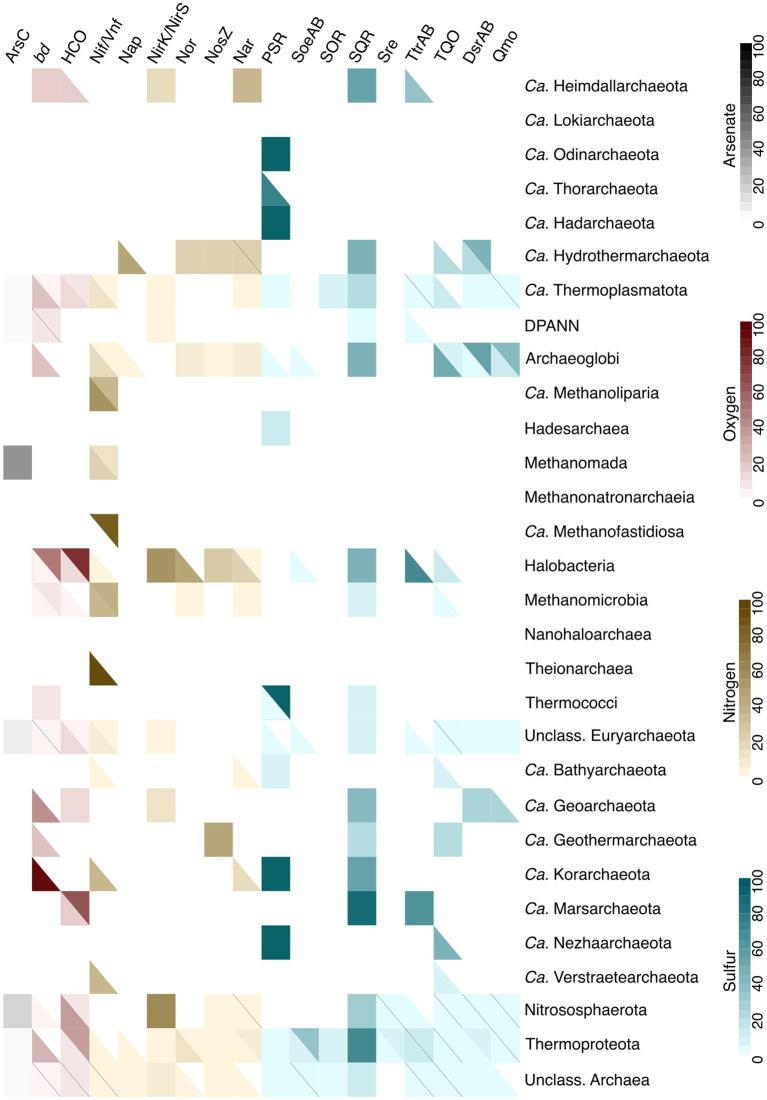
Presence of the marker proteins for different types of energy metabolism (KO and DiSCo annotations). Arsenate detoxification is marked in grey, oxygen metabolism in red, nitrogen metabolism in yellow, and sulfur metabolism in blue. ArsC: arsenate reductase, bd: bd oxidase, HCO: heme-copper oxidase, Nif/Vnf: nitrogenase, Nap: nitrate reductase, NirK/NirS: nitrite reductase, Nor: nitric oxide reductase, NosZ: nitrous oxide reductase, Nar: nitrate reductase, PSR: polysulfide reductase, SoeAB: sulfite:quinone dehydrogenase (subunits A and B), SOR: sulfur oxigenase/reductase, SQR: sulfide:quinone oxidoreductase, Sre: sulfur reductase, TtrAB: tetrathionate reductase (subunits A and B), TQO: thiosulfate:quinol oxidoreductase, DsrAB: dissimilatory sulfite reductase (subunits A and B), Qmo: quinone-modifiyng oxidoreductase. A full solid colored square indicates that, if a marker gene is a multisubunit complex, all of the subunits are present in a certain percent of the genomes. In cases where a square is split into two colors, the top part of the square indicates the percentage of genomes containing a full complex, while the bottom part shows the percent of genomes that have incomplete complexes.

Various types of energy metabolism were investigated using gene markers for arsenic, nitrogen, oxygen and sulfur metabolism (Figure 7). Our findings indicate that organisms capable of detoxifying arsenate include *Methanomada,* as well as unclassified *Euryarchaeota* and *Nitrososphaerota*. Regarding oxygen metabolism, both *bd* oxidases and heme-copper oxidases (Pereira et al., 2001) were detected in *Ca.* Heimdallarchaeota, *Ca.* Thermoplasmatota*, Halobacteria, Methanomicrobia, Ca.* Geoarchaeota, *Nitrososphaerota, Thermoproteota,* unclassified *Euryarchaeota*, and unclassified Archaea. Some lineages have genes that encode only *bd* oxidase (DPANN, *Archaeoglobi, Thermococci, Ca.* Korarchaeota, *Ca.* Geothermarchaeota), while others have only HCO genes (*Ca.* Marsarchaeota). However, many of the hits did not pass the cutoffs, even lowered cutoffs, such as the case for HCOs in *Ca.* Heimdallarchaeota, where, despite earlier evidence of presence of these enzymes in this specific lineage (Spang et al., 2019; Bulzu et al., 2019), only one out of five genomes recovered a partial HCO complex.

Six marker proteins/complexes were selected to cover the diversity of nitrogen metabolism, although not including the ammonia monooxygenase AmoA, which shares a KO with the methane monooxygenase PmoA (K10944), and hence they are difficult to differentiate (Holmes et al., 1995). The hits for K10944 were nonetheless found in the dataset, in *Nitrososphaerota* (ammonia-oxidizers; Pester et al., 2011), as expected. The distribution of the Nif nitrogenase, used as protein marker for nitrogen fixation, recovered a similar distribution to that described in Baker et al. (2020), being found in *Ca.* Thermoplasmatota, *Methanomicrobia*, and *Theionarchaea*. However, in our case, additional 11 lineages had hits for nitrogenases, such as *Archaeoglobi*, *Ca.* Methanoliparia*, Methanomada*. Possible explanations for this difference could be the inclusion of vanadium-dependent nitrogenase Vnf in our results, or our search for all Nif subunits, as compared to Baker et al. (2020) using only NifH as a marker. Other cases, such as nitrite reductases NirK/NirS, did not overlap. For example, none of the lineages analyzed in Baker et al. (2020) were reported to contain NirS, and only *Aigararchaeota* and *Nitrososphaerota* were said to contain NirK. However, while our dataset did not include *Aigarachaeota*, other lineages had a hit for NirK in our dataset (e.g., *Ca.* Heimdallarchaeota, *Ca.* Thermoplasmatota*, Thermoproteota*), and NirS was found in *Halobacteria* (a lineage not included in Baker et al., 2020 analysis). It is possible, however, that additional NirK hits are in fact false positives due to the NirK homology to multicopper oxidases (Bento et al., 2005; Solomon et al., 1996).

To cover dissimilatory sulfur oxidation and reduction in archaea, seven protein markers were selected, ranging from sulfur oxygenase reductase (SOR; Urich et al., 2004; Urich et al., 2006) and thiosulphate:quinone oxidoreductase (TQO; Müller et al., 2004), first characterized in *Acidianus ambivalens* and representing chemolithoautotrophic sulfur-oxidizing metabolism in *Thermoproteales* (*Sulfolobales*, *Acidobales*), to the DsrAB and Qmo proteins to mark the Dsr-dependent dissimilatory sulfate/sulfite reduction in Archaea. The results recapture the known diversity within this dataset (Neukirchen and Sousa, 2021; Anantharaman et al., 2018; Friedrich et al., 2001; Ghosh and Dam, 2009). However, some of the newly discovered archaeal lineages with metabolic potential for Dsr-dependent sulfur metabolism, such as *Ca. Methanodesulfokores washburnensis* (McKay et al., 2019) or Dsr-containing Aigarchaeota (Hua et al., 2018) are absent from our dataset, explaining why this metabolism was not found in those groups. On the contrary, sulfide:quinone oxidoreductases were found to be present across 16 different archaeal groups, such as *Ca.* Heimdallarchaeota, *Halobacteria*, *Ca.* Korarchaeota, and *Thermoproteota*. So far, archaeal Sqrs have only been characterized from *A. ambivalens* (Brito et al., 2009) and *C. maquilingensis* (Lencina et al., 2013), and due to their sequence homology with Ndh-II (Brito et al., 2009), it cannot be excluded that some of these results are false positives, and the distribution of Sqr in Archaea is, in fact, smaller.

Using gene markers for terminal oxidoreductases or central complexes to pinpoint metabolic traits, while effective in uncovering the potential for certain types of energy metabolism in Archaea, falls short of presenting a comprehensive view of the possible variability within energy metabolic strategies. This approach may overlook the emergence of novel complexes formed through the rearrangement of modular protein components into unique architectures, not accounted for in these types of analyses.

## 4 Discussion

The aim of this paper was straightforward: to conduct a large-scale investigation into what is known and what is yet to be discovered within the archaeal domain, and to assess how much of archaeal metabolism can be reconstructed automatically using computational approaches. However, this turned out to be a much more complex analysis than initially thought, due to the biases of knowledge regarding the other two domains of life, the different pathways of Archaea, and the fact that, with the exponential increase in sequencing projects and discovery of new lineages, their sequence divergency (real or due to sequencing artifacts) cannot be scaled up/incorporated in real time to existing databases. It is well-known that most of the current biological knowledge is based on Bacteria and Eukaryotes, with little attention given to incorporating Archaea and their differences into metabolic modules and pathways. Archaeal metabolism and information processing can be different from the ones present in Bacteria and Eukaryotes, and archaeal unique biochemical pathways enables them to thrive in extreme environments and utilize diverse substrates, often relying on coenzymes and cofactors that necessitate entirely different enzymatic reactions compared to bacterial metabolic pathways. One prominent example is the incorporation of selenocysteine (Stadtman, 1974), often referred to as the 21st amino acid (Böck et al., 1991), that is found in proteins from the three domains of life (Rother and Quitzke, 2018) Selenocysteine is synthesized via a complex mechanism involving a specific tRNA and a dedicated set of enzymes (Chambers et al., 1986), and the archaeal synthesis is more related with the eukaryotic than with the bacterial one (Rother and Quitzke, 2018). This amino acid plays a crucial role in the function of several selenoenzyme families, including glutathione peroxidases and thioredoxin reductases, which are vital for oxidative stress management and redox reactions in archaeal cells (Rother and Quitzke, 2018). Another noteworthy cofactor found in a small number of methylamine-metabolizing archaea as well as a few bacteria (Tharp et al., 2018; Hao et al., 2002; Srinivasan et al., 2002; Borrel et al., 2014) is pyrrolysine, traditionally known as the 22nd amino acid. Pyrrolysine is encoded by the UAG codon in some methanogenic Archaea (Hao et al., 2002; Srinivasan et al., 2002), and is integral to the activity of methyltransferase enzymes (Soares et al., 2005), which are involved in the final steps of methane production from methylated compounds (Rother and Quitzke, 2018). The existence of pyrrolysine highlights the diversity of genetic codes and implications for protein synthesis in archaea, further underlining their unique metabolic capabilities. Moreover, coenzymes such as coenzyme M (2-mercaptoethanesulfonic acid) and coenzyme F_430_ are central to the metabolic pathways of methanogens and other anaerobic archaea (Kaster et al., 2011), albeit also being found in some bacterial organisms. Coenzyme F_430_, a nickel-containing porphyrin, plays an essential role in the enzymatic reaction catalyzed by methyl-coenzyme M reductase, where it participates in the final step of methane production, showcasing a highly specialized enzymatic system (Thauer et al., 2008). These unique biochemical components reveal how archaea have evolved distinct metabolic strategies that not only allow them to occupy a wide range of ecological niches but also highlight the evolutionary divergence between archaea and other life forms.

These differences, as well as the usage of non-archaeal sequences in the modules can lead to misassignments or false negatives in terms of functional predictions. For example, HCOs from *Ca.* Heimdallarchaeota fall short of the cutoffs for homology-based annotation. It is possible that this lineage’s proteins diverge significantly from those in reference databases. However, this issue is not unique to *Ca.* Heimdallarchaeota; it also applies to halobacterial HCO proteins, indicating that not all divergence can be explained by this alone. Here we have shown 37.6% of the archaeal protein space remains uncharacterized, and that over 96% of archaeal metagenomes contain long stretches of genes, for which not even the protein domains (PFAM) are known. Also, within the uncharacterized proteins with PFAM annotations available, many contain cofactors and metal centers thought to have been playing a pivotal role since the origin of Life (Sanchez-Rocha et al., 2024; Weiss et al., 2016). For these uncharacterized cofactor-containing enzymes, the function is not yet known, and they may be a part of archaeal specific unexplored pathways, whose characterization would increase the diversity of microbial biology.

Enhancing current genomic classification databases and functional predictive models may involve refining them through additional analyses, like synteny analysis or integrating other omics data. This approach requires more sophisticated knowledge and operations rather than simple clicks to access and interpret this information. Some progress has been made regarding sulfur metabolism, where several dedicated tools, such as HMSS2 (Tanabe and Dahl, 2023) or DiSCo (Neukirchen and Sousa, 2021), were carefully built to identify specific types of metabolism, already integrating the current microbial diversity known. Progress has also been made in developing annotation-free strategies for identification of microbial dioxygen utilization from reads data, and in the last years, methods for TF identification from gene-expression data from quantitative phenotyping analysis (Darnell et al., 2017), approaches for a systematic inference of TF activity (Ma and Brent, 2021) and computational models for topological comparison of regulatory networks across the two domains of Life (Robinson and Schmid, 2018) have been developed. Another expanding area is phenomics, with several tools being developed in the last years, such as MicroPIE (Mao et al., 2016) to enable a fast extraction of phenotypic information from text records. Recently, the Functional Annotations of Prokaryotic Taxa (FAPROTAX) database (Louca et al., 2016) was tested for fast-functional screening of microbial metabolism, based on 16S RNA data (Sansupa et al., 2021) with promising results.

However, for an in-depth analysis of large datasets, better and faster tools need to be developed. Here is where statistical information theory (IT) plays an important role. Methods such as Mutual Information (MI; Vinga, 2014), Distance Correlation and its variants (Szé Kely and Rizzo, 2013; Monti et al., 2023) that already are useful to analyze, e.g., gene expression matrices, should be further developed to allow, e.g., comparisons of gene expression levels and inferences across independent samples. Moreover, in a recent study, MI was employed for pathway analysis, and, when applied to single-cell data, yielded robust and meaningful scores (Jeuken and Käll, 2024). For sequence data, IT can provide a broad range of inferences, from TF binding sites to gene mapping and phenotypic predictions, as comprehensively reviewed by Vinga (2014).

Artificial intelligence (AI), particularly machine learning algorithms, can also, in principle, provide valuable insights into archaeal metabolism by analyzing large genomic datasets and by filling in some of the gaps (Hoarfrost et al., 2022). Machine learning can aid in genome annotation (Chen et al., 2024; Khodabandelou et al., 2020), predict enzyme functions (Salas-Nuñez et al., 2024 and references within), and reconstruct metabolic pathways (Libbrecht and Noble, 2015). However, challenges and limitations persist in the field and the accuracy of metabolic reconstructions relies heavily on the quality of genomic and biochemical data available for archaeal species. Missing data and heterogenous datasets can lead to severe overfitting and other problems, as heavily discussed in the literature (Rodrigues, 2019; Xu and Jackson, 2019; Altman and Krzywinski, 2018). Customized models that consider the unique features of archaeal genomes and metabolic pathways can improve the accuracy and specificity of reconstructions. Integrating genomic, transcriptomic, proteomic, and metabolomic data can provide a comprehensive view of archaeal metabolism. AI and machine learning approaches that combine and analyze multi-omics data will facilitate more accurate reconstructions and deeper insights into the metabolic capabilities of Archaea, especially if coupled to statistical information theory. Perhaps this is the way to go in the future. But we must remember that computers only see zeros and ones (much better than we do), so we cannot forget that biology is more than math, and that without proper constraints and curated training data from experimental characterizations, distinguishing real results from artifacts is an almost impossible task. Moreover, as we have shown in this paper, without human manual curation, and extensive literature searches to get experimental archaeal characterized proteins to fill gaps in pathways, the distance between the vast amount of genomic information available and their analysis will only increase. In a perfect world, all data would be of high quality, with consistent information across different platforms. Additionally, linguistic and other barriers would be reduced to unite experimentalists, microbiologists, computational scientists, and mathematicians in the shared goal of closing this gap. Joining bottom-up with state of the art top-down predictive (ML) and inference (IT) approaches—merging the “*in silico*” and “*in vivo/vitro*” could increase the speed at which we explore the archaeal world and disentangle its mysteries. This strategy would increase our understanding of archaeal metabolism, and life in general, offering new insights and opportunities for further research.

## Data Availability

The files with full annotation, all taxonomic levels for uncharacterized counts, and synteny tables, can be found at Figshare: 10.6084/m9.figshare.25782123. The pipeline (incl. examples of arCOG analysis scripts and plotting scripts) is available on GitHub: https://github.com/valkaravaeva/protein_classification_tool.
