# Supplementary material for: Navigating the archaeal frontier: insights and projections from bioinformatic pipelines
Source: Front Microbiol. 2024 Sep 23;15:1433224. doi: 10.3389/fmicb.2024.1433224 (PMC11459464; doi:10.3389/fmicb.2024.1433224)
Supplement: Supplementary file 1 [file Data_Sheet_1.PDF]

Supplementary Material

Navigating the Archaeal Frontier: Insights and Projections from Bioinformatic Pipelines

Val Karavaeva[1,2], Filipa L. Sousa[1*]

[1]Genome Evolution and Ecology Group, Department of Functional and Evolutionary Ecology, University of Vienna, Vienna, Austria

[2] Vienna Doctoral School of Ecology and Evolution, University of Vienna, Vienna, Austria

*** Correspondence:**
Filipa L. Sousa filipa.sousa@univie.ac.at

1 Supplementary Figures and Tables

Supplementary Tables 1-7 are uploaded as separate files.

This supplementary material includes:

Supplementary Figures 1-4

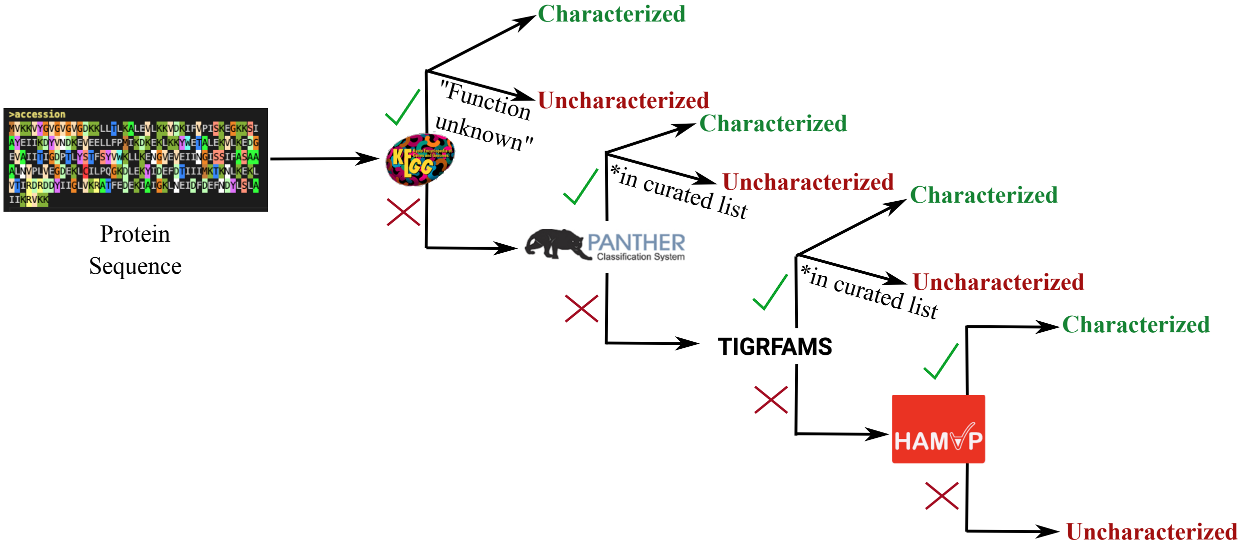

Supplementary Figure 1. *The pipeline scheme to classify proteins as "characterized" or "uncharacterized" (as described in Materials and Methods).*

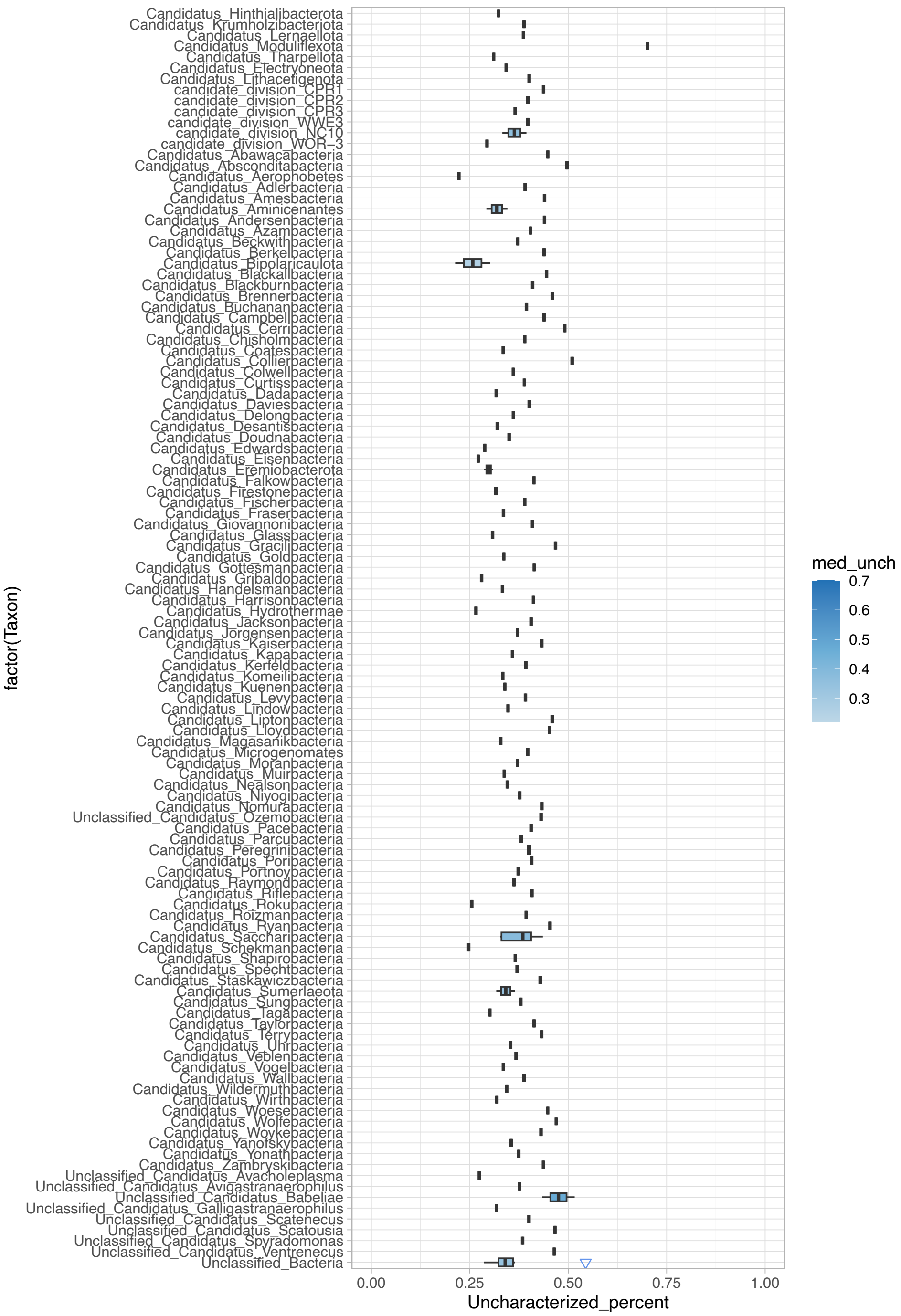

Supplementary Figure 2. *Percentage of unclassified bacterial proteins for Candidate phyla according to pipeline classification (see Materials and Methods) per phylum. For complete taxonomic information see Suppl. Table 1. For exact percentages, see Suppl. Table 4.*

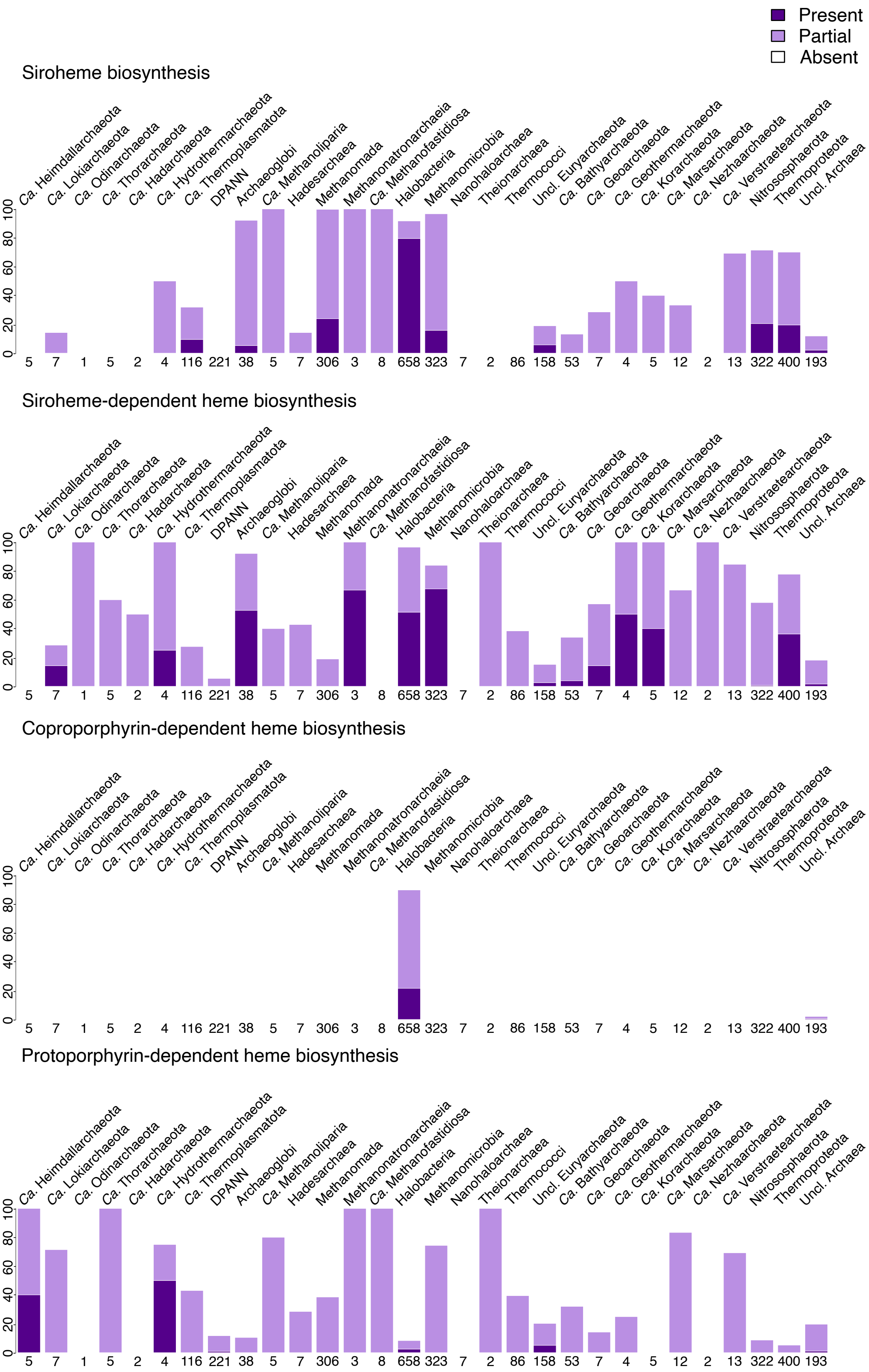
Present
Partial
Absent
Siroheme biosynthesis
Siroheme-dependent heme biosynthesis
Coproporphyrin-dependent heme biosynthesis
Protoporphyrin-dependent heme biosynthesis
Ca. Heimdallarchaeota
Ca. Lokiarchaeota
Ca. Odinarchaeota
Ca. Thorarchaeota
Ca. Hadarchaeota
Ca. Hydrothermarchaeota
Ca. Thermoplasmatota
DPANN
Archaeoglobi
Ca. Methanoliparia
Hadesarchaea
Methanomada
Methanonatronarchaeia
Ca. Methanofastidiosa
Halobacteria
Methanomicrobia
Nanohaloarchaea
Theionarchaea
Thermococci
Uncl. Euryarchaeota
Ca. Bathyarchaeota
Ca. Geoarchaeota
Ca. Geothermarchaeota
Ca. Korarchaeota
Ca. Marsarchaeota
Ca. Nezhaarchaeota
Ca. Verstraetearchaeota
Nitrososphaerota
Thermoproteota
Uncl. Archaea
0 20 40 60 80 100
5 7 1 5 2 4 116 221 38 5 7 306 3 8 658 323 7 2 86 158 53 7 4 5 12 2 13 322 400 193

Supplementary Figure 3. *Presence of cofactor biosynthesis in archaea (per phylum for most, per class for Euryarchaeota, with Methanobacteria, Methanococci, and Methanopyri grouped into the Methanomada supergroup) based on modified KEGG modules. From top to bottom: presence of siroheme biosynthesis, presence of siroheme-dependent heme biosynthesis, presence of coproporphyrin-dependent heme biosynthesis, presence of protoporphyrin-dependent heme biosynthesis. Dark purple indicates that the full module is present, light purple marks the presence of the incomplete module, white shows the absence of the module in a lineage.*

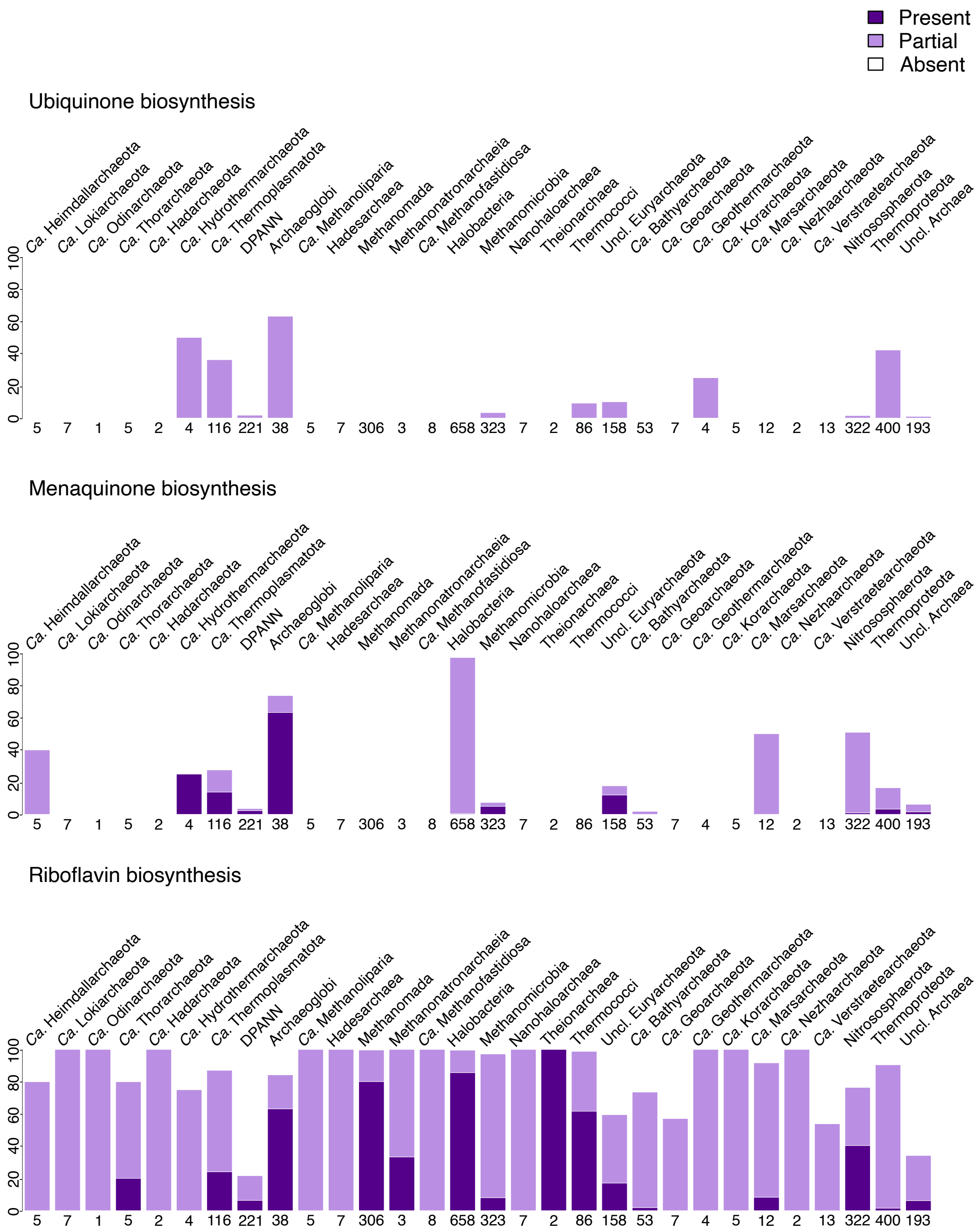

Supplementary Figure 4. *Presence of cofactor biosynthesis in archaea (per phylum for most, per class for Euryarchaeota, with Methanobacteria, Methanococci, and Methanopyri grouped into the Methanomada supergroup) based on modified KEGG modules. From top to bottom: presence of ubiquinone biosynthesis, presence of menaquinone biosynthesis, presence of riboflavin biosynthesis.*

Dark purple indicates that the full module is present, light purple marks the presence of the incomplete module, white shows the absence of the module in a lineage.
